# Potential Future Applications of Postbiotics in the Context of Ensuring Food Safety and Human Health Improvement

**DOI:** 10.3390/antibiotics14070674

**Published:** 2025-07-03

**Authors:** Zorica Tomičić, Ljubiša Šarić, Ružica Tomičić

**Affiliations:** 1 Institute of Food Technology in Novi Sad, University of Novi Sad, Bulevar cara Lazara 1, 21000 Novi Sad, Serbia; ljubisa.saric@fins.uns.ac.rs; 2 Faculty of Technology, University of Novi Sad, Bulevar cara Lazara 1, 21000 Novi Sad, Serbia

**Keywords:** postbiotics, natural bioactive compounds, functional food, clinical application, anti-inflammatory, antimicrobial, antioxidant activity

## Abstract

Postbiotics are defined as non-viable metabolites or compounds produced by probiotic microorganisms with significant impact on human health. The growing interest in postbiotics is supported by numerous studies due to their additional benefits over probiotics that show positive outcomes for specific conditions, as well as their application as biopreservatives in the food industry. Their potential in functional foods and therapeutic applications is increasingly recognized as they exhibit stability, safety, and diverse biological activities. As for their most important biological roles, postbiotics have been shown to have effective anti-inflammatory, antimicrobial, antioxidant, and anticancer properties, in addition to reducing food allergies. The application of postbiotics in functional foods contributes to improving intestinal health and reducing the risk of foodborne diseases. The concept of postbiotics is relatively new in the food industry. They offer a promising alternative to conventional food preservatives due to their ability to inhibit pathogenic bacteria and extend shelf life. Considering the diversity of postbiotic compounds and their significant biological activities, this review presents and discusses the mechanisms of action and future trends in their application in the food industry and their impact on human health. Increasing research and development in the production and formulation of postbiotics will play a key role in the upward trajectory of the market.

## 1. Introduction

Antibiotics have a long history of application in the treatment of bacterial infections, which are responsible for numerous diseases in humans and animals. However, their inappropriate and excessive use has led to an increase in bacterial resistance to antimicrobial drugs and to an imbalance in the host microbiota. Alerted to this crisis, the World Health Organization (WHO) launched a global action to combat antimicrobial and antibiotic resistance in May 2015. Despite this, the rate of resistance among various bacterial species such as *Staphylococcus* spp., *Pseudomonas* spp., *Enterobacteria* spp., *Enterococcus* spp. and *Acinetobacter* spp. continues to increase [1,2]. Bacterial resistance to antimicrobial agents has become a growing threat to global health, with a wide impact on humans, and is predicted to rise and cause 10 million deaths each year by 2050 [3,4]. The European Community has recently invested large amounts of funds into research on antimicrobial resistance, confirming its social and medical importance [1]. Therefore, alternative approaches with direct therapeutic treatment of the diseases are crucial. Among these, it has been shown that probiotics and postbiotics can help restore the balance of the intestinal microbiota, modulate the immune system, and act against pathogens. In addition, consumer demands for healthier and more natural foods emphasize the importance of studying natural substances to ensure food safety [5]. Considering the above, we will further clarify the main mechanisms of postbiotic action that provide health benefits, and their potential application in the food industry to improve food safety and shelf life.

Probiotics are well known as “good microorganisms” and are officially defined as the live microorganisms that, when administered in adequate amounts, confer a health benefit on the host. Most probiotics are the bacterial species of *Lactobacillus* and *Bifidobacterium* and the yeasts *Saccharomyces* spp., which are generally considered safe (GRAS) and whose main function is to maintain intestinal health and regulate the immune system [5]. Through their antagonistic properties, probiotics help to establish a microenvironment in the intestine that favors beneficial microorganisms and reduces the colonization of pathogens through competition for nutrients and binding sites. They produce bioactive metabolites such as bacteriocins, hydrogen peroxide, antimicrobial peptides, and organic acids, and modulate the function of the host immune system [2,6]. One of the important mechanisms of action is the modulation and regulation of intestinal immune responses by reducing proinflammatory cytokines, increasing the secretion of IgA production, improving intestinal barrier function and increasing mucin synthesis. Microorganisms intended for probiotics must meet certain criteria, such as survival in gastrointestinal conditions while withstanding low pH, organic acids, and enzymes present in the intestine, being able to adhere to the intestinal mucosa, being non-pathogenic and non-toxic, and being stable under storage and field conditions [5]. Their beneficial properties are well known, such as preventing and treating acute diarrhea, lowering blood cholesterol and blood pressure, reducing intestinal inflammation, strengthening the immune system, preventing cancer, reducing food allergies, helping in the production of vitamins B and K, and preventing the growth and proliferation of invasive bacteria [5,6,7].

Probiotics, as live microorganisms, can offer various health benefits, such as enhancing the function and health of the host intestine, supporting beneficial bacteria, and potentially inhibiting harmful ones. Recently, components produced from probiotics, namely postbiotics, have emerged as examples of bioactive compounds that can be used to inhibit the proliferation of pathogenic microorganisms as innovative antimicrobial agents. Although the exact definition of postbiotics is debated, the International Scientific Association for Probiotic and Prebiotics (ISAPP) defines it as “a preparation of inanimate microorganisms and/or their components that confers a health benefit on the host”. Postbiotics are the complex mixture of metabolites produced by probiotics or cell wall fragments, such as organic acids, enzymes, amino acids, peptides, fatty acids, antimicrobial peptides, polysaccharides, etc. In general, postbiotics have shown all the beneficial effects of probiotics without side effects in experimental and preclinical studies [8,9,10]. They possess numerous advantages including anti-inflammatory, antioxidant, and immunomodulatory, and show significant efficacy in addressing obesity. Due to their safety, they may be a superior alternative to the live probiotic cells from which they are produced, due to their defined chemical composition, stability over a wide range of temperatures, broad spectrum of antimicrobial activity, and ease of use and storage, and they have significant applications in the food and pharmaceutical industries. This means that their health benefits do not rely on the activity of live cells, and are therefore not subject to the strict food safety regulations that govern live microorganisms [7,11]. However, current and future market trends for postbiotics, knowledge gaps, and applications in medicine and the food industry require further analysis and consideration.

Novel technologies, methods, and applications currently being investigated demonstrate that postbiotics have nutritional and general health benefits. Their use as functional ingredients may have advantages in foods that are considered detrimental to the survival of probiotics [5,10]. Industrial application as an alternative to conventional food preservatives may have significant advantages, as they inhibit the growth of harmful bacteria and thus extend the shelf life of food [12]. Despite these developments, there are still many uncertainties regarding their health properties and bioactivities. Therefore, this review aims to encompass the latest aspects of research into the role of these bioactive compounds as promising agents for ensuring food safety and improving human health.

## 2. Postbiotic Mechanisms of Action

**Figure 1:**
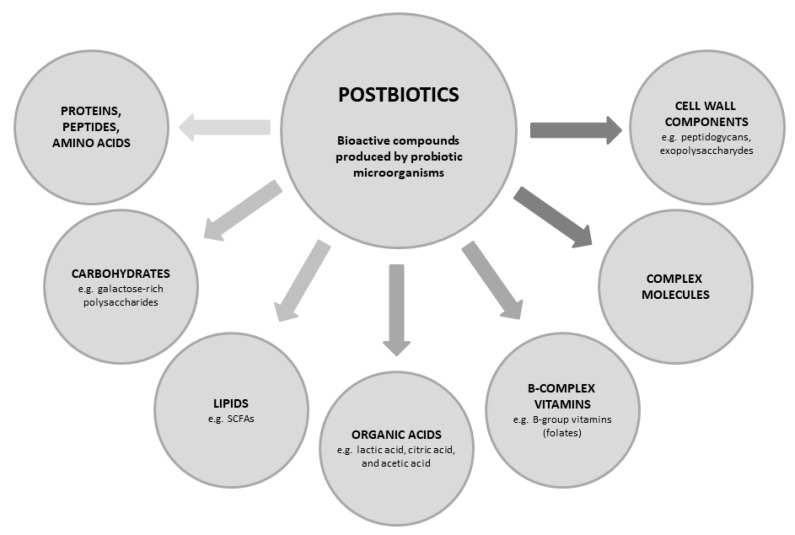
Main postbiotics with health promoting effects.

Postbiotics are a diverse group of bioactive compounds that can be broadly classified based on their structure, elemental composition, or physiological function. They include proteins (e.g., lactocepin), carbohydrates (e.g., teichoic acids and galactose-rich polysaccharides), lipids (e.g., acetate, butyrate, dimethyl acetyl-derived plasmalogen, lactate, propionate), organic acids (e.g., propionic), B-complex vitamins, and other complex molecules (Figure 1). Postbiotics as novel therapeutic agents show promising potential with multiple mechanisms of action on human health that are largely not clearly understood, but there is growing evidence that these compounds exert their effects through various biological pathways. They enhance innate immunity, reduce pathogen-induced inflammation, and enhance intestinal barrier functions [7,8,9,13]. Several well-known properties of postbiotics include prevention of infection, antitumor and antioxidant activity, immunomodulation, and lipid/cholesterol metabolism [14].

*Lactobacillus* spp., as one of the most widely studied probiotic microorganisms, are a rich source of antibacterial postbiotics that limit the growth and activity of specific pathogens, contributing to the maintenance of host intestinal health. They have been shown to improve intestinal permeability through several additional mechanisms, restoring optimal levels of tight junction proteins and factors involved in mucin production [15,16]. Other postbiotics, such as exopolysaccharides secreted from *Lactobacillus plantarum,* protect intestinal epithelial cells by promoting goblet cell differentiation, which produces mucus [17]. Postbiotics contribute to wound healing by improving epithelial proliferation and differentiation [15]. Moreover, they possess anticancer activity and increase apoptosis in cancer cells [18]. Postbiotics produced by *Lactobacillus rhamnosus* GG and *Bacteriodes fragilis* have the ability to increase levels of IL-10, IgA and IFN-γ, which play a key role in immune responses and are important cytokines in reducing inflammatory processes [8,19]. Lipoteichoic acid as a postbiotic exhibits immunomodulatory properties depending on the strain from which it is produced. It has been shown to reduce the secretion of pro-inflammatory cytokines, while increasing the production of the anti-inflammatory cytokine IL-10. In addition, it inhibits the expression of the mitogen-activated protein kinase (MAPK) and nuclear factor kappa-B (NF-κB) pathways, crucial for the inflammatory response induced by lipopolysaccharide. It also has the potential to target the multifactorial pathogenesis of metabolic dysfunction-associated steatotic liver disease (MASLD) [8].

Postbiotics such as butyrate, a short-chain fatty acid, can stimulate the production of regulatory T cells in the intestine. These cells help control the immune response by suppressing the activity of other T cells to prevent overactivity, which can cause autoimmune diseases [20]. With its anti-inflammatory potential, butyrate may serve as a therapeutic agent for patients with inflammatory bowel disease. On the other hand, the results of clinical trials to date have varied, ranging from significant efficacy for patients, to studies showing no effect on intestinal inflammation [21,22]. One reason for these contradictory effects may be the dose or formulation. High doses of butyrate have been shown to induce cell cycle arrest and apoptosis, while low doses below 2 mM stimulate colonocyte proliferation [23]. However, further research suggests that postbiotics, such as butyrate, may relieve symptoms in people with mild to moderate ulcerative colitis or Crohn’s disease, two types of inflammatory bowel diseases, by activating immune cells that reduce inflammation [24]. Postbiotics, such as cell wall fragments and supernatant from probiotic gut bacteria, can increase the production of anti-inflammatory cytokine molecules, which help reduce inflammation and strengthen the body’s immune response [25,26].

## 3. Biological Activities of Postbiotics

**Figure 2:**
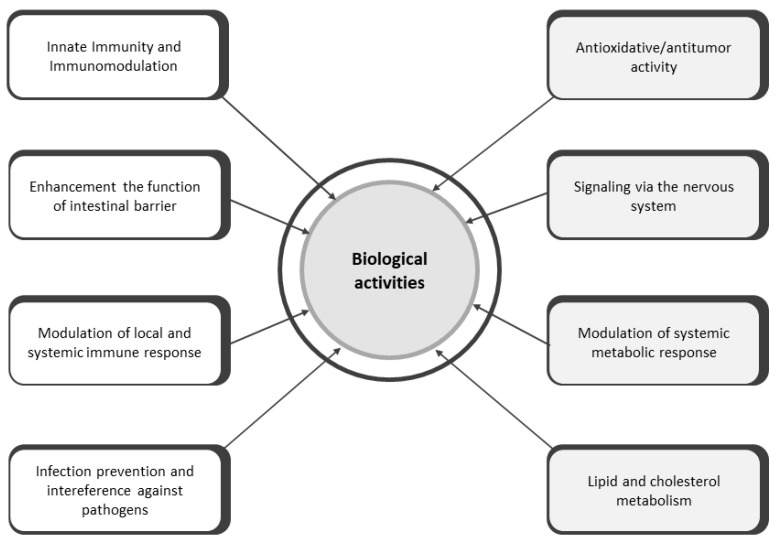
Bioactive properties of postbiotics.

Postbiotics, with their known chemical structures and safe doses, can be a safe alternative to probiotics [9]. Clinical and experimental studies have not yet identified any adverse effects, which has resulted in their increased use in the prevention and treatment of certain diseases due to bioactive properties such as immunomodulatory, anti-inflammatory, antioxidant, anti-cancer, and antimicrobial properties (Figure 2). Certain medical approaches are promising in the treatment of cancer through various mechanisms such as enhancing the host immune response, improving the gastrointestinal microflora, inducing apoptosis, and having antiproliferative effects [27,28]. Despite all the advantages mentioned above, they face numerous challenges in clinical application due to insufficiently clear and specific mechanisms of action, interactions with the host and other substances, as well as standardization of production. In addition, different strains of microorganisms produce different postbiotic compounds, whose specific composition significantly affects their biological activity. Therefore, further research is needed to confirm their efficacy and to develop methods for their delivery and application [8].

### 3.1. Anticancer Activity

Anticancer activity is largely reflected in their ability to bind with host immune cells, activating certain signaling pathways and leading to an increase in the innate immune system response and a decrease in inflammation [29]. A study performed by Chuah et al. [27] reported the cytotoxicity of six postbiotics derived from *L. plantarum* strains against various malignant cancer cells, which were strain- and dose-dependent, with an inhibition rate of up to 50%. Similarly, the exopolysaccharide from *L. plantarum* 70810 from Chinese Paocai demonstrated anticancer activity against colon carcinoma cells [30], while cell wall protein fractions from *Lactobacillus paracasei* showed apoptotic responses by reducing the cell growth of a human colon carcinoma cell line [31]. Further clinical studies are needed to optimize postbiotic delivery approaches and their potential post-oral treatment, as well as their ability to induce apoptosis and inhibit cancer cell proliferation.

### 3.2. Antioxidant Activity

While postbiotics are known for their various health benefits, their antioxidant properties require more consideration and research. Oxidative stress can lead to inflammation, cell damage, and increased susceptibility to diseases in the body, requiring a search for natural sources of antioxidants [32]. It has been shown that metabolites of *L. plantarum* can reduce protein and lipid oxidation through the mechanisms of hydroxyl radical scavenging (HRS) and reducing power (RP). The high antioxidant potential of *L. plantarum* 15 cells and the cell-free supernatant assessed by the DPPH method reached 82.65% and 72.21%, respectively [33]. Chang et al. [34] reported positive correlations between organic acid production and antioxidant activity exhibited by postbiotics produced from *L. plantarum* strains. In this study, the growth of *L. plantarum* strains in formulated media with increased concentrations of yeast extracts containing amino acids and growth factors promoted the production of postbiotic metabolites with enhanced biological functionalities, such as antimicrobial and antioxidant activities. According to Liu et al. [35] and Wang et al. [36], exopolysaccharides excreted from the probiotic cultures *Lactobacillus fermentum* and *Paenibacillus polymyxa* showed strong antioxidant effects, and may be a therapeutic option in diseases such as diabetes, rheumatoid arthritis, and atherosclerosis. Inhibitory activity against various pathogens is achieved by the production of bacteriocins and organic acids, namely acetic acid, caproic acid, and lactic acid [34]. Among the carbon sources in formulated media, glucose has been shown to be the best ingredient for bacteriocin production compared to other carbon sources such as sucrose, due to its simple structure [37]. Due to their diverse qualities, bacteriocins are widely used in various applications, including medicine, food, cosmetics, the pharmaceutical industry, and cancer therapy.

### 3.3. Antiviral Potential

Postbiotics have a wide range of bioactivities and a potential for use in biomedical and pharmaceutical applications due to their antioxidant, anti-inflammatory, antitumor, immunomodulatory and antibacterial activities. As an additional benefit, they also have antiviral potential, which contributes to public health where viral infections are a global problem. They exert an antiviral effect by limiting the replication of viruses through the induction of pro-inflammatory immune responses and the production of inflammatory cytokines and interleukins, such as TNF-α, IL-23, IL-18 and IL12, and the cytotoxic activation of T-lymphocytes, NK cells and monocytes/macrophages [1]. Postbiotics from *Lactobacillus amylovorus*, *L. plantarum*, and *Enterococcus hirae* have shown antiviral potential against enterovirus isolates recovered from acute flaccid paralysis cases. The highest susceptibility to the cell-free supernatant of *L. plantarum* was observed for EV19, with inhibition percentages of 51.8% and 60.9% for pre- and post-treatment, respectively [38].

### 3.4. Anti-Inflammatory Potential

Anti-inflammatory functions in the host intestine can be enhanced by the secretion of postbiotics such as bacteriocins, amino acids, short-chain fatty acids, vitamins, etc. Numerous advantages in maintaining intestinal barrier functions and repairing epithelial wounds in the host can be also achieved through the induction of anti-inflammatory cytokines such as transforming growth factor beta (TGF-β) and interleukin 10 (IL-10) [13]. Clinical studies of the anti-inflammatory potential of the heat-treated postbiotic strain *Bifidobacterium longum* CECT-7347 have shown that it is effective in protecting the intestinal barrier and opens avenues for expanding its application to maintain intestinal health. It further exhibits protective capacity against oxidative stress damage, inhibits bacterial colonization, and activates pathways associated with innate immune function [39]. Short-chain fatty acids have shown a variety of health effects in the host. In addition to improving function and blood flow in the colon, they also promote epithelial cell proliferation [40]. In controlling inflammatory conditions, the functional activity of postbiotics depends on several factors, especially the type of postbiotic strain. Multi-strain postbiotics have been shown to possess more potent anti-inflammatory potential compared to single-strain postbiotics [13].

## 4. Antimicrobial Role of Postbiotics

The increasing demand for food safety, combined with a focus on the preservation of nutritional values and quality has stimulated a large amount of research into finding natural antimicrobial agents and applying them in the food industry [41]. Postbiotics have advantages over antibiotics and chemical preservatives in inhibiting pathogenic and food spoilage bacteria. They exhibit notable antimicrobial properties that stem from various bioactive compounds that are effective in inhibiting the growth of food-borne pathogenic bacteria such as *Bacillus cereus*, *Salmonella enterica*, and *Staphylococcus aureus* [7,42]. They possess the ability to influence bacterial behavior by disrupting their cell structure, interfering with quorum sensing, and inhibiting nutrient uptake, thereby preventing infection and biofilm formation. The antimicrobial effect of postbiotics depends on a number of factors such as the concentration of the postbiotic, the target bacteria (since Gram-negative are generally more resistant to postbiotic compounds than Gram-positive, because of the presence of the additional protection afforded by the outer membrane), and the nature of the original prebiotics involved [9,10,25]. Hydrogen peroxide is the main metabolite of lactic acid bacteria, whose antibacterial effect depends on its concentration and is manifested by strong oxidative functions on the bacterial cell and damage to cytoplasmic protein structures [43].

Previous studies have shown that organic-acid based postbiotics have significant antimicrobial potential. Among the most important organic acids produced by probiotic bacteria are citric, tartaric, and acetic acids, which have a strong antibacterial effect. The role of acetic acid, tartaric acid, citric acid, and malic acid in inhibiting pathogens is to reduce intracellular pH, while lactic acid reduces membrane integrity [7,44]. Furthermore, high concentrations of organic acids and low pH can inhibit the growth of spoilage and disease-causing microorganisms [45]. A study conducted by Tong et al. [46] examined a novel postbiotic produced by *Bacillus amyloliquefaciens* J and *L. plantarum* SN4, and showed that water extract of postbiotics (PWE) exhibited potent antimicrobial activity against three common foodborne pathogens (*Escherichia coli* O157: H7 ATCC 43889, *S. aureus* ATCC 43300, and *Salmonella typhimurium* ATCC 14028) with the minimal inhibitory concentration (MIC) ranging from 18.75 mg/mL to 25 mg/mL. Lactic acid and acetic acid were identified as the main antibacterial components in PWE.

Bacteria play a key role in the complex process of synthesis and production of a wide range of peptides, among which ribosomal and non-ribosomal types of antimicrobial peptides are distinguished [24]. Bacteriocins obtained from different *Lactobacilli* and *Bifidobacteria* strains are peptides or proteins that exhibit potent antimicrobial activities and have been used in fermented foods for years. Their antibacterial mechanism is reflected through action on the cytoplasmic membrane, forming pores and inhibiting the growth and development of pathogens. Other advantages include their resistance to heat and pH [47,48]. Unlike traditional antibiotics, bacteriocins produced by *Lactobacilli* act bacteriostatically or bactericidally by targeting the bacterial cell envelope and specific species without affecting other populations within the same ecosystem [49]. In a study conducted by Zhang et al. [50], bacteriocins produced by *L. plantarum* LPL-1 isolated from fermented fish showed a significant inhibitory effect against *Listeria monocytogenes*, acidifying the cell membrane and creating pores in the bacterial membrane. The ribosomally synthesized bacteriocin showed potential in inhibiting biofilm formation of food borne and pathogenic bacteria by disrupting cell–cell interactions, loss of quorum sensing signals, and downregulation of virulence factors. In the last few decades, bacteriocins have received increasing attention as potential next-generation antimicrobial drugs to reduce the threat of diseases caused by drug-resistant pathogens, and as possible immunomodulatory agents. However, there is a barrier to their widespread use in the food industry and medicine due to insufficient data on toxicity and safety, which is why numerous studies are being conducted [24].

The antibacterial mechanisms of fatty acid-based postbiotics include destabilization of the bacterial cell membrane by increasing permeability and causing cell lysis, which consequently inhibits bacterial cell growth. In addition, they disrupt the electron transport chain process and interfere with oxidative phosphorylation, two of the most important vital processes of the cell membrane and essential for energy production in cells. Moreover, they can directly inhibit membrane enzymatic activity and nutrient uptake [7,51]. Eicosapentaenoic acid (EPA) is active against Gram-positive bacteria [52], while lauric and myristic acids inhibit microbial growth and development [53].

In the food matrix, probiotic bacteria produce large amounts of vitamins, especially in dairy products, while in the host intestine, vitamin production is significantly lower. Vitamin C has the greatest antimicrobial potential, increasing the acidity of bacterial cell membrane lipids, which leads to lysis of the cell membrane and the bacterial cell wall [43].

## 5. Postbiotics in Food Application

Increased consumer awareness of nutritional and health values has sparked interest in the field of functional foods, where postbiotics as bioactive compounds are playing an increasing role. As beneficial by-products of microbial fermentation, they can be found naturally in a variety of fermented foods like yogurt, pickled vegetables, sauerkraut and kombucha. These bioactive compounds are produced by various bacterial and fungal species such as *Lactobacillus*, *Bifidobacterium*, *Streptococcus*, *Saccharomyces boulardii*, *Eubacterium*, *Faecalibacterium*, etc. [10]. On the other hand, there are different methods for their preparation, including heat treatment, enzymatic treatment, ultrasonication, high-pressure processing, and filtration techniques, which are intended to extract them from microbial cells. These techniques offer significant advantages such as effective microbial inactivation, preservation of valuable bioactive compounds, enhanced release of intracellular components, and the high purity of extracted substances. The choice of technique must be adapted to each bacterial strain, to ensure the highest yield and functionality of the bioactive compounds [9,54]. Their use as functional food ingredients during the handling and commercialization of food products offers numerous advantages over the bacteria from which they are produced due to their stability and safety, which has contributed to the growth of the functional food market. Furthermore, they do not require cold chains in industrial use for transportation and storage as probiotic products, do not interact with the food matrix or affect the change in taste or odor, and there is also no possibility of developing antibiotic resistant genes [14]. Although the precise impact on health and host immune response is still unknown, scientific evidence supports their antibacterial, antioxidant, anti-inflammatory, and immunomodulatory properties. Owing to their ability to treat certain diseases and improve health, their supplementation may help in the prevention and treatment of gut-related diseases such as symptoms of inflammatory bowel disease and irritable bowel syndrome [8,10].

In the food industry, postbiotics are used to develop functional foods with the aim of ensuring the safety of these foods, thereby improving nutritional values and human health. Postbiotics help prevent the growth of harmful bacteria, which contributes to reducing food spoilage and extending the shelf life of products, making them an alternative to conventional food preservatives. As an additional benefit, due to their stability over a wide temperature and pH range, they can be added to foods and ingredients before thermal processing with preserved functional properties. Table 1 provides a summary of the applications of postbiotics in food products and their benefits. Exopolysaccharides produced by *Lactobacillus* spp. play a significant role in dairy products, which can contribute to improving the sensory and physicochemical quality of the product [12]. Among the tested concentrations in the range of 0.05% to 0.15% (w/w), inclusion of 0.10% polysaccharide extracts from *Lactarius volemus* Fr. in yogurt achieved the best sensory characteristics of the product. On the other hand, its presence influenced the increase of essential amino acids and viable probiotic bacteria cells in probiotic yogurt [55]. Another study showed that the postbiotic supernatant from *Lactobacillus plantarum* YML007 can serve as a bio-preservative and contribute to extending the shelf life of soybeans [56]. Bacteriocins have shown significant potential in food preservation. Among them, nisin is the first bacteriocin to receive regulatory approval for commercial use as a preservative by the European Food Safety Authority (EFSA) and the Food and Drug Administration (FDA) [14]. It has found application in numerous foods such as dairy products, infant formula, and canned soups [57].

Microbial contamination of meat and fish products can significantly alter their nutritional value and taste, making them unsafe for consumption. However, coating and spraying techniques with postbiotic extracts of these products have proven to be a promising option for their preservation [62]. Moradi et al. [58] demonstrated that cell supernatants from *Lactobacillus salivarious* were effective in controlling microbial growth in ground beef samples, exhibiting notable antimicrobial activity against psychrotrophic spoilage bacteria at 4 °C. In other studies, postbiotics from *Lactobacillus rhamnosus* EMCC 1105 completely inhibited *Clostridium perfringens* on minced meat during storage at 6 °C [59], while bacteriocins from *Bifidobacterium lactis* Bb-12 extended the shelf life of minced meat during cold storage by reducing the number of *Aeromonas* and *Pseudomonas* spp. [9]. As antimicrobial agents, postbiotics have shown inhibitory effects against coliform bacteria, aerobic mesophilic bacteria, and molds in fruits and vegetables. Postbiotics from *L. plantarum* and *Lactobacillus acidophilus* extended the shelf life of vegetables by reducing the growth of pathogenic bacteria *Escherichia coli* and *S. aureus*, and molds *Aspergillus niger* and *Aspergillus flavus* [60]. Their application in food offers numerous advantages such as extending the shelf life of food, reducing the risk of pathogen transmission through the food chain, providing additional protection during excessive exposure to temperature, better preservation of food nutritional value and organoleptic properties, and reducing the use of chemical preservatives [47,61]. All of this points to the significant role of postbiotics as biopreservatives in meat, fish, fruits, and vegetables and their products.

Effective prevention of microbial biofilms in the food industry is a major global challenge. Bacterial cells within biofilms exhibit high resistance to antimicrobials, harsh environmental conditions, and host immune defenses, making them difficult to control and treat. Therefore, preventive approaches must be focused on the development of functional antimicrobial agents, which include effective natural compounds [63]. The application of postbiotics as antibiofilm agents can help prevent biofilm formation or destroy developed biofilm on surfaces and materials in the food industry. They can prevent bacteria from adhering to surfaces and forming initial aggregates, key steps in biofilm formation, as well as disrupt quorum sensing (QS) systems that bacteria use to communicate and form biofilms. Among them, bacteriocins, polysaccharides, and biosurfactants are postbiotics with antibiofilm properties [62,64]. Postbiotics of *Lactobacillus* spp. have been shown to possess effective antibiofilm activity. Previous studies have confirmed the ability of postbiotics produced from *Lactobacillus* spp. to eliminate *L. monocytogenes* and *S. aureus* biofilms, which activity depended on the contact time and type of postbiotic [58,65]. A study conducted by Moradi et al. [58], investigates the potential of postbiotics derived from *Lactobacillus* species to inhibit *L. monocytogenes*, both in vitro and in food models. The results showed that the most potent postbiotics in preventing biofilm formation of *L. monocytogenes* were obtained from the *Lactobacillus salivarius* strain, with an inhibition rate of 88% compared to *L. acidophilus* and *L. casei*. Postbiotics not only alter biofilm formation, but also disrupt and eliminate it through various mechanisms of action, such as damaging the bacterial membrane and targeting genes involved in biofilm formation [66]. In addition, their effectiveness can be influenced by internal and external factors, such as the chemical composition of the food matrix (enzymes) and storage conditions related to pH and temperature. Therefore, to ensure their optimal antibiofilm effect in food products, encapsulation strategies and integration of postbiotics into the structure of packaging films can be applied [66,67]. Although studies have shown the potential of postbiotics to disrupt biofilm formation and even degrade existing biofilms, translating these findings into effective applications in various industries requires further research and validation.

## 6. Future Perspectives of Postbiotics

Postbiotics play an important role in the human body and have unique benefits over probiotics that are demonstrated by a large amount of scientific evidence. They have shown numerous advantages in terms of safety, distribution in the body, and technological and economical properties. After being defined by ISAPP, the postbiotics market continues to take shape and has a wide scope for development and for attracting the food and pharmaceutical industries [8]. However, there are numerous challenges, including limited understanding of their long-term effects and potential inconsistencies in efficacy, their classification between dietary supplements and medical products, and standardized production processes that make it difficult to establish clear regulatory frameworks for their development and commercialization. The establishment of clear regulatory frameworks and quality assurance protocols is essential to ensure that postbiotics meet safety and efficacy standards [68]. For industrial-scale production, progress is required in several areas, including optimized cultivation of the probiotics from which they are produced in fermentation systems, the development of efficient and cost-effective methods for the extraction and purification of postbiotics, as well as increased formulation stability and delivery efficiency [67]. Their use in nutrition may have significant therapeutic applications, especially in populations suffering from specific diseases. Although postbiotics show promise for various health benefits, understanding how they interact with the human body at a molecular level is still an area of active research [10,15]. With a notable antimicrobial potential, postbiotics can serve as an alternative to existing drugs and help combat antimicrobial resistance through various mechanisms, including modulating the gut microbiota, strengthening the intestinal barrier, and stimulating the immune response. Postbiotics play a significant role in food safety, extending the shelf life of food products by controlling the growth of pathogens and spoilage microorganisms, and maintaining sensory properties [42]. In industrial applications, they may be more reliable than probiotics, because many factors such as pH, water activity, processing and storage conditions, and nutritional composition can affect the survival of probiotics [9]. Among the bacteriocins, nisin has been used for food preservation and is GRAS [14]. Although still a new idea in the food and pharmaceutical sector, these probiotic-derived substances represent potential for future applications.

## 7. Conclusions

This review offers a comprehensive description of postbiotics, bioactive natural agents as alternatives to antibiotics, with a discussion of some evidence for their beneficial uses for human health protection and application in the food industry through the results of recent research. Undoubtedly, postbiotics possess numerous advantages over the probiotics from which they are produced, due to their defined chemical composition, safety, stability over a wide range of pH and temperature, ease of use, and storage. To date, studies of postbiotic molecules are still in their infancy, and the mechanisms of actions have not yet been elucidated. Research indicates that postbiotics can help overcome metabolic syndromes and produce immune signals to establish defense systems at the cellular level. With their antimicrobial potential, postbiotics have a strong inhibitory effect against various enteropathogens, and they neutralize their toxins in the intestinal system. Furthermore, they can significantly affect the reduction of anti-inflammatory and autoimmune disorders, and they also exhibit cytotoxic effects on cancer cells. However, there is a need for more clinical trials to confirm their benefits and understand their interactions with the body. The role of postbiotics in the field of food and nutrition is promising in terms of the development of new products and their application in packaging systems as biopreservatives in the food industry. Their non-toxicity, safety, and ability to inhibit pathogenic microorganisms, extend shelf life, and potentially improve overall gut health are crucial features for their application. It is necessary to fully understand how postbiotics impact human health and to optimize their application in the food industry.

## Figures and Tables

**Table 1:** Postbiotics in food products and their benefits.

Postbiotic	Food	Benefits
Polysaccharide extracts from *Lactarius volemus* Fr.	Yogurts	Improvement in water-retention capacity and reduction in pH; extended shelf life [55]
Supernatant from *Lactobacillus plantarum* YML007	Soybeans	Extended shelf life [56]
Nisin	Dairy products, infant formula, canned soups	Acts as a preservative [14,57]
Pirrolo [1,2-a] and pyrazine-1,4-dione from *Lactobacillus salivarious*	Ground meat and whole milk	Antibiofilm activity against *Listeria monocytogenes* [58]
Cell-free supernatants from *Lactobacillus rhamnosus* EMCC 1105	Poultry meat	Inhibition of *Clostridium perfringens* [59]
Bacteriocins from *Bifidobacterium lactis* Bb-12	Minced meat	Inhibition of *Aeromonas* and *Pseudomonas* spp., extended shelf life [9]
Cell-free supernatants from *Lactobacillus plantarum* Cs and *Lactobacillus acidophilus* ATCC 314	Tomato paste	Inhibition of *Staphylococcus aureus*, *Escherichia coli*, *Aspergillus niger* and *Aspergillus flavus*; extend shelf life [60]
Exopolysaccharide from *Lactobacillus rhamnosus*	Cheddar cheese	Improvement of product performance [61]

## Data Availability

The original contributions presented in the study are included in the article; further inquiries can be directed to the corresponding author.
